# Current methods of diagnosis and treatment of scaphoid fractures

**DOI:** 10.1186/1865-1380-4-4

**Published:** 2011-02-04

**Authors:** Steven J Rhemrev, Daan Ootes, Frank JP Beeres, Sven AG Meylaerts, Inger B Schipper

**Affiliations:** 1 Department of Trauma Surgery, Medical Centre Haaglanden, The Hague, The Netherlands; 2 Department of Trauma Surgery, Leids University Medical Centre, Leiden, The Netherlands

## Abstract

Fractures of the scaphoid bone mainly occur in young adults and constitute 2-7% of all fractures. The specific blood supply in combination with the demanding functional requirements can easily lead to disturbed fracture healing. Displaced scaphoid fractures are seen on radiographs. The diagnostic strategy of suspected scaphoid fractures, however, is surrounded by controversy. Bone scintigraphy, magnetic resonance imaging and computed tomography have their shortcomings. Early treatment leads to a better outcome. Scaphoid fractures can be treated conservatively and operatively. Proximal scaphoid fractures and displaced scaphoid fractures have a worse outcome and might be better off with an open or closed reduction and internal fixation. The incidence of scaphoid non-unions has been reported to be between 5 and 15%. Non-unions are mostly treated operatively by restoring the anatomy to avoid degenerative wrist arthritis.

## Introduction

The carpal scaphoid bone is known to play a key role in the function of the wrist. Therefore, pathologic abnormalities of the scaphoid may have serious consequences. Scaphoid fractures account for 2-7% of all fractures and predominantly occur in young, active males. Of all carpal fractures, 82-89% concern scaphoid fractures. The incidence in Western countries is approximately five fractures in every 10,000 inhabitants [1-3]. However, because of the diagnostic challenge that scaphoid fractures often present, the exact incidence is unknown.

Given the above, the indistinct method of treatment and the tremendous research efforts over the last decade resulting in up to 3,200 PubMed hits, the scaphoid remains one of the most interesting carpal bones for researchers.

### Anatomy

The scaphoid fracture was first described in 1905 by Destot, a French surgeon, anatomist and radiologist [4]. The word scaphoid is derived from the Greek word for boat (skaphos). Because of its unique anatomy it can articulate with all five surrounding bones (distal radius, os capitatum, os lunatum, os trapezium and os trapezoideum).

Eighty percent of the scaphoid bone consists of cartilage, leaving limited space for entrance of the supplying arteries. The main blood supply is through retrograde branches of the radial artery. The dorsal branch of the radial artery provides 75% of the blood supply through the foramina. The palmar branch reaches the scaphoid via the distal tubercle. Contrary to the proximal pole, the distal pole and the tubercle have an independent vascularisation. The proximal pole depends on blood supply from the distal pole through the scaphoid bone. In case of a proximal scaphoid fracture, the blood supply through the scaphoid bone is interrupted, making the healing process of the proximal pole particularly more difficult [5].

### Clinical presentation

The typical trauma mechanism is a fall on the outstretched hand with the wrist in radial deviation inducing impact of the palm. This trauma mechanism also puts the dorsal radius and the scaphoid-lunatum (SL) ligament at risk. The above-described mechanism causes the scaphoid bone to impact against the distal radius concavity, causing a fracture most likely to occur in the middle of the scaphoid. There is an increased chance of a proximal pole fracture when falls occur on the wrist in abduction [6].

Interestingly, the same trauma mechanism causes supracondylar humeral fractures in children and distal radius or carpal fractures in the elderly [7].

**Figure 1:**
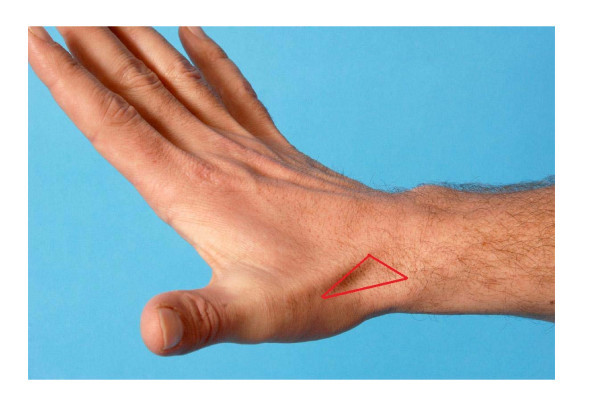
**The anatomic snuffbox**.

There are no reliable clinical tests to confirm or rule out the diagnosis of a scaphoid fracture. An observable swelling of the anatomic snuffbox (Figure 1) increases the chance of a scaphoid facture. Pain when applying pressure on the anatomic snuffbox or the scaphoid tubercle, or when applying axial pressure on the first metacarpal bone all have a sensitivity of 100%. However, their specificity is 9%, 30% and 48%, respectively [8]. Other studies found a higher specificity for a tender tubercle (57%). An over 50% diminished grip strength compared to the contralateral side increases the positive predictive value for a scaphoid fracture [9,10].

## Imaging of the scaphoid

There are several different diagnostic modalities to detect a scaphoid fracture. These include conventional radiographs, computed tomography (CT scans), magnetic resonance examination, bone scintigraphy and sonograms. Each procedure has its specific advantages and disadvantages (Table 1).

**Table 1:** Sensitivity and specificity for bone scans, MR examination and CT scans

	Sensitivity (%)	Specificity (%)
Bone scan [19]	100 (83-100)	90 (81-96)
MR examination [19]	80 (56-94)	100 (96-100)
CT [48]	93 (83-98)	99 (96-100)

### Conventional radiographs

**Figure 2:**
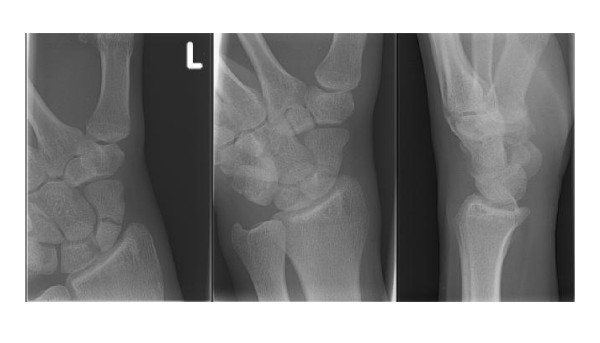
**Initial radiograph (patient A): a postero-anterior view; b oblique view; c. lateral view**.

Scaphoid fractures are often missed with the use of conventional radiographs alone. Initial radiographs (Figure 2) detect at most 70% of all scaphoid fractures [11]. There is still no consensus regarding the different types of conventional radiographs. Anterior-posterior and lateral radiographs should be standard, and at least two additional views are advocated for a suspected scaphoid fracture [12].

Even on the repeated radiographic exam after 10-14 days propagated by many clinicians in case of an occult fracture, a scaphoid fracture is often missed, since the additional sensitivity is low, although in case of sclerosis it could confirm the suspected diagnosis [13-15].

### Computed tomography (CT)

**Figure 3:**
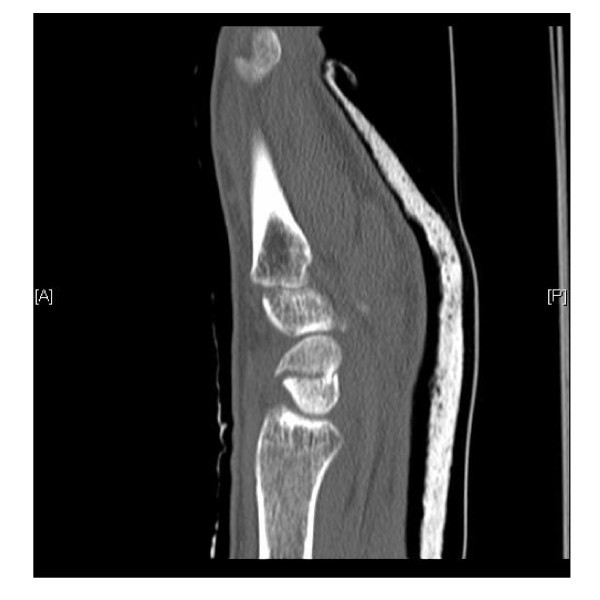
**CT scan (patient B) sagittal view of a fractured scaphoid**.

The costs and radiation exposure for a computed tomography (Figure 3) scan are comparatively low. CT is readily available in both hospitals and emergency departments, which enables CT confirmation of a suspected scaphoid fracture. CT imaging also allows adequate judgement of cortical involvement and is therefore often used in the decision-making process concerning whether or not to operate on scaphoid fractures.

Unfortunately, the sensitivity of CT is lower in comparison to bone scintigraphy [16]. A solid statistical statement about the CT as a diagnostic tool for scaphoid fractures is difficult to make because of insufficient inclusion of patients in research to date. Despite the high resolution and multiplanar reconstructions, the difficulty of the interpretation of a CT scan lies in the distinction between channels in the trabecular bone pattern and fractures. This restricts the specificity of the CT scan [16-18].

### Bone scintigraphy

**Figure 4:**
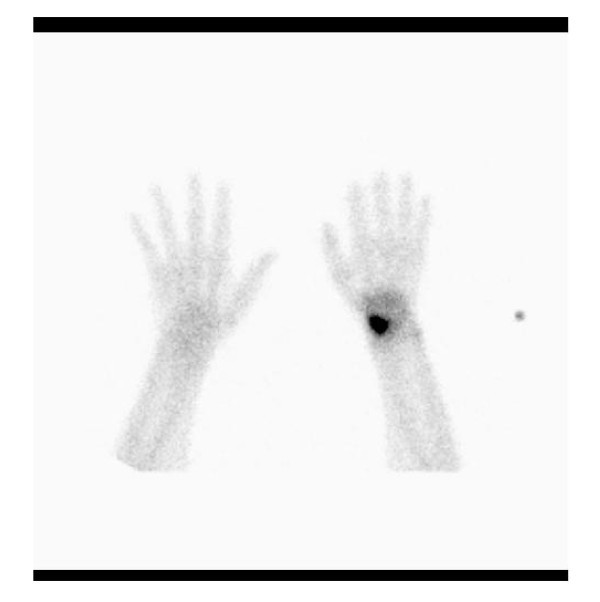
**Bone scintigraphy (patient C) of the hands the patient with a scaphoid fracture on the right side**.

Using a bone scan (Figure 4), scaphoid fractures can be ruled out with a high level of confidence. For this reason it is recommended as a second diagnostic modality of choice after conventional radiographs. The sensitivity is close to 100%, whereas the specificity depends on the modality that is defined as the gold standard for comparison. Bone scintigraphy results in up to 25% false-positive outcome measures [15]. The procedure is reliable and relatively fast, but patients have to pay an extra visit to the hospital, and it requires intravenous radioactive isotopes. In addition, bone scintigraphy is expensive [19-21].

### Magnetic resonance examination

**Figure 5:**
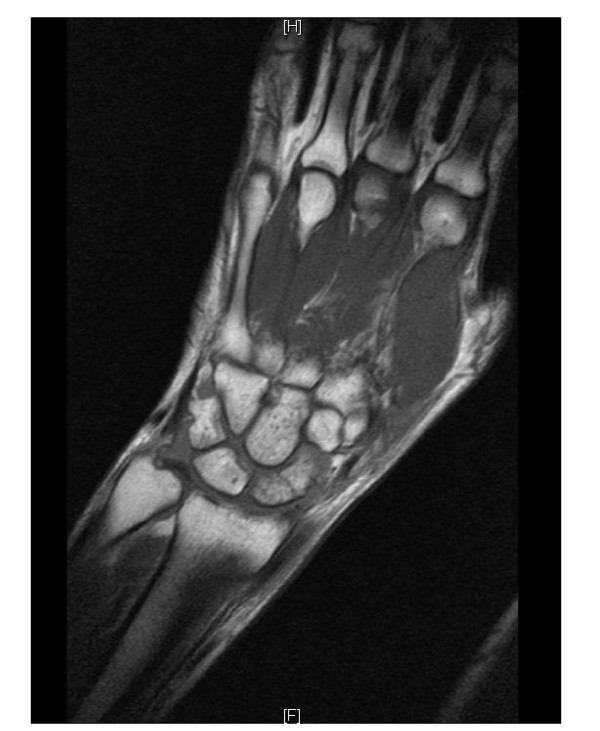
**Magnetic resonance imaging (patient D) of a waist fracture of the scaphoid**.

Magnetic resonance (MR) examination is often recommended as a diagnostic modality for occult scaphoid fractures (Figure 5) [22,23,13]. Late MR examination (after 19 days) shows better results in comparison to bone scintigraphy in terms of sensitivity and specificity [20]. However, the early MR imaging within 1 day after trauma has a limited sensitivity of 80% [19]. The interpretation of a MR examination depends strongly on the experience of the clinician. When adequately performed, MR examination enables simultaneous diagnosis of soft tissue and ligament injuries. Considerable experience is needed for the distinction between swelling and oedema, micro-fractures or incomplete fractures, or complete but nondislocated fractures. The MR examination also has infrastructural restrictions. Not every hospital has an MR scan, and if available there are often many structural and organizational problems to overcome.

### Sonogram

The routine use of ultrasound is not indicated to diagnose a scaphoid fracture. Low-frequency ultrasound has not proven to be of any advantage, whereas high-frequency ultrasound can be helpful in the diagnosis of a scaphoid fracture. The interpretation of ultrasound is again dependant on the level of experience of the clinician. The use of ultrasound in the diagnostic process of an occult scaphoid fracture is still subject to research and therefore not yet established as a useful standard diagnostic modality [24,25].

In conclusion, a gold standard with a positive predictive value of 100% for scaphoid fractures does not currently exist. Routine radiographs at baseline are mandatory, and repeated radiographs are not indicated to detect occult scaphoid fractures. Univocal data regarding the advocated diagnostic tool for imaging suspected scaphoid fractures are still limited.

## Classification of scaphoid fractures

Many classifications are used for carpal scaphoid fractures. Three will be discussed here in order of their clinical relevance.

### Herbert classification

The Herbert classification [26] is based on the stability of the fracture. Unstable fractures are fractures with a dislocation of more than 1 mm or an angulation of more than 15° between the fragments. Additional fractures, trans-scaphoid-perilunate dislocations, multi-fragment fractures and proximal pole fractures are also classified as unstable.

### MAYO classification

**Figure 6:**
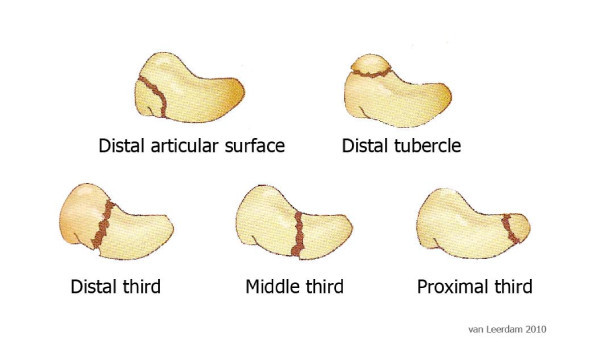
**MAYO classification for scaphoid fractures**.

**Figure 7:**
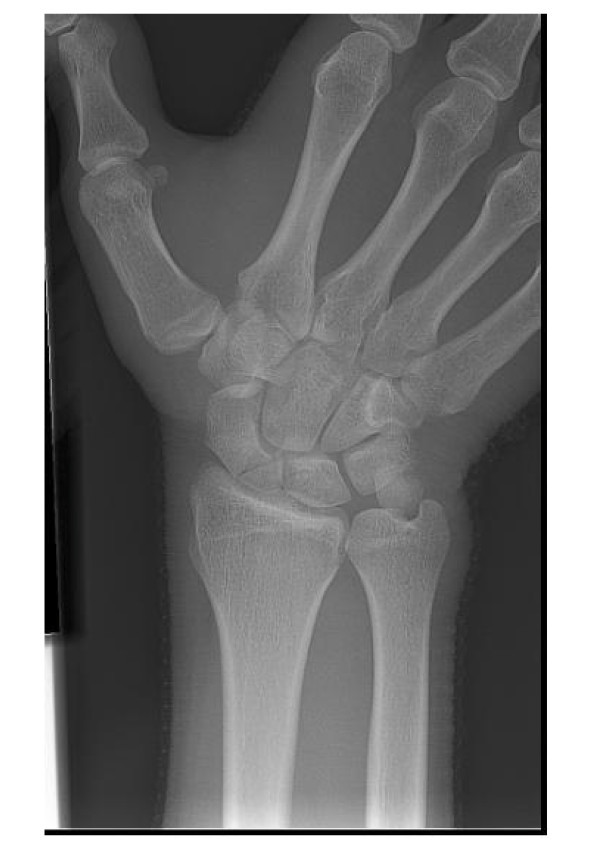
**Proximal pole fracture (patient E) on a conventional radiograph**.

The MAYO classification [27] (Figure 6) divides scaphoid fractures into proximal (Figure 7) (10%), middle (70%) and distal (20%) fractures. Within the distal third, distinction is made between the distal articular surface and the distal tubercle.

### Russe classification

The anatomic classification according to Russe [28] predicts the tendency of the fracture to heal. The classification distinguishes among horizontal oblique, transverse or vertical oblique fracture lines. The vertical oblique fracture is unstable, whereas the horizontal oblique and the transverse fractures are more stable fractures.

## Treatment

The aim of the treatment is to achieve fracture consolidation and functional recovery whilst avoiding complications such as non- or mal-union. Therapeutic options consist of direct functional treatment, cast immobilisation of the fracture and joints, and operative treatment.

### Direct functional treatment

The literature shows that a scaphoid fracture can be treated functionally. In case of a clinically suspected scaphoid fracture without radiological signs of a fracture, early functional treatment can be started using a bandage or an orthosis. Patients with persistent clinical suspicion of a scaphoid fracture should have repeated radiological evaluation within 7 days after the trauma to evaluate the current treatment strategy and to potentially adjust the treatment strategy as a result based on the radiographic findings.

Inadequate immobilisation of a scaphoid fracture increases the chances for pseudo-arthrosis by 30% [29-31]. We therefore believe that there is no indication to treat a proven scaphoid fracture functionally without cast immobilisation or operative fixation.

### Cast immobilisation

In case of an occult or stable scaphoid fracture according to the current Herbert classification, cast immobilisation is still the therapy of choice.

Scaphoid fractures are hard to immobilise, since nearly every motion of the hand, wrist and forearm causes movement of the bone and pressure on the fracture line. Therefore, even an "above the elbow" cast may be applied [32].

There are different types of cast immobilisation for a scaphoid fracture either with or without inclusion of the thumb. There is no study proving a better consolidation with regard to the type of cast that is used; however immobilisation in slight dorsal extension seems to have a positive effect on the grip strength and range of motion of the wrist joint [33-35].

**Figure 8:**
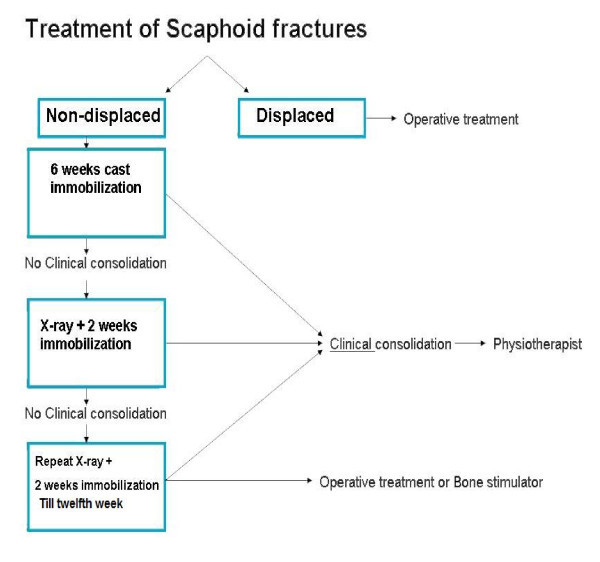
**Treatment of scaphoid fractures**.

The duration of immobilisation varies, depending on the type of fracture and the outcome on repeated radiological check-ups, which serve as an estimation of fracture consolidation. Generally, a cast treatment of 6 weeks (Figure 8) should be sufficient in most non-displaced and stable fractures [36]. Cast immobilisation has been proven to be a reliable and successful treatment with low costs and a low complication rate.

### Operative treatment

**Figure 9:**
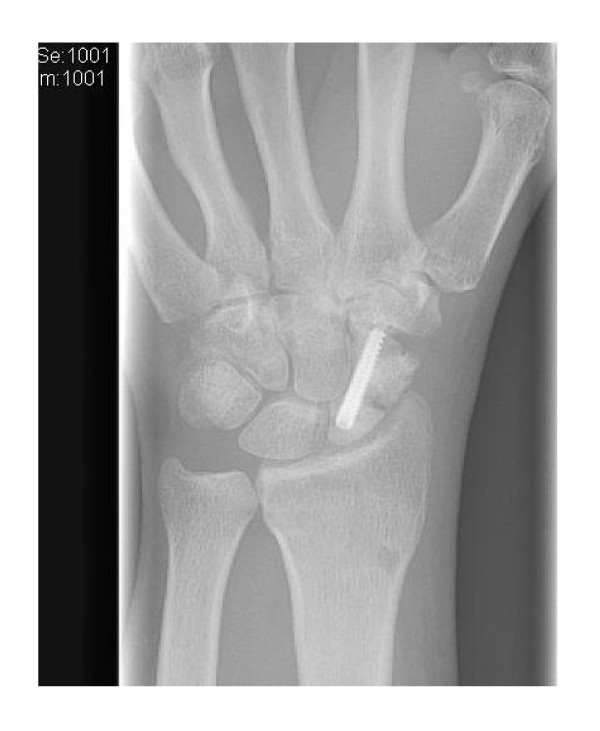
**X-ray after percutaneous screw fixation of the scaphoid (patient F)**.

With improved, minimally invasive surgical techniques, surgical treatment of non-displaced scaphoid fractures has increased. The advantage of operative management with percutaneous screw fixation (Figure 9) in a non-displaced fracture is the possibility of early functional treatment [37-39].

Operative treatment is indicated in unstable fractures according to the Herbert classification. However, there is no uniformity of opinion on the operative treatment of a non-dislocated fracture of the proximal pole. The scaphoid bone can be approached both from dorsal and volar directions. Distal and middle fractures are best approached from the volar side because of good exposure and conservation of the blood supply. Displaced proximal pole fractures require a dorsal approach because accurate placement of the screw will then be easier to perform. Because of the improved minimally invasive surgical techniques with limited trauma, an increase in surgically treated patients has evolved [40,38]. In this manner, a prolonged immobilization period of often 8-12 weeks can be prevented. Wrist stiffness and reduced wrist strength were less frequently observed if a surgical procedure was successful. Moreover, the demand for strategies that allow early productivity of the young patient and the relatively high cost of prolonged immobilization have contributed to the shift towards surgical interventions. There is, however, still insufficient evidence concerning which treatment is preferable for the non-displaced scaphoid fracture [37].

### Complications

Both conservative and operative treatment may cause complications. These include delayed union, osteonecrosis, pseudo-arthrosis and the related instability, arthrosis and collapse of the carpal joint. These complications may result in serious functional restrictions with regard to mobility and grip strength. Additional complications in case of an operation are malalignment, failure to place the screw, re-operation, infections and soft tissue injuries.

In case of a delayed union of the scaphoid fracture, a bone stimulator or magnetic field therapy can be used to achieve bone union [41]. Medicinal treatments are also described. However, evidence-based data are limited, and therefore this treatment is not generally accepted [42].

Pseudo-arthrosis often remains asymptomatic, and may become evident and symptomatic in case of a new trauma or in case of excess strain of the wrist joint. Pseudo-arthrosis in case of an operative treatment depends on the type of fracture and varies between 5 and 50% [43,44]. A symptomatic pseudo-arthrosis is best treated operatively. Anatomical fracture reduction and intra-articular alignment will prevent an early arthrosis. Several operational techniques have been described. These always include debridement, realignment and implementation of a native vascularised or non-vascularised bone grafting, with or without the use of osteosynthesis [45,46,28]. The success rate of this procedure is between 74 and 94%. In case of proximal pseudo-arthrosis, the results are much worse [47]. There are no prospective randomized clinical trials that compare vascularised and non-vascularised bone grafting.

Arthrosis can be a late complication of a scaphoid fracture. A sustainable reduction of pain and functional improvement are often no longer achieved in such cases. The so-called rescue operations in case of arthrosis are styloidectomy, denervation of the carpal joint, and the total or partial removal of the scaphoid with four-quadrant fusion (lunate bone, triquetral bone, capitate bone and hamate bone).

Very few evidence-based data exist regarding the treatment of and diagnostic modalities for scaphoid fractures.

Scaphoid non-union remains a difficult problem. Early recognition and improvement in treatment will decrease the incidence of this problem and will avoid late complications.

## Competing interests

No funds were received in support of this study.

S.J. Rhemrev, M.D.

Steven Rhemrev has been a trauma surgeon since 2001. He attended the University of Amsterdam Medical School in 1985. In 1995 he started his training for General Surgery at the Free University Medical Centre Amsterdam under the supervision of Prof. Dr. Haarman. In the past years he has continued his residency in Trauma Surgery at Medical Centre Alkmaar. He specialised in Traumatology at the VU Medical Centre with Prof. Dr. Patka and Prof. Dr. Haarman (2001-2003). He received a fellowship in Orthopaedic Trauma at Zams, Austria, and at the Liverpool Trauma Centre in Sydney, Australia. From 2003 to the present he has been working at the Medical Centre Haaglanden, which is a level 1 trauma centre in The Hague, The Netherlands, as a surgeon specialised in Trauma Surgery. He is the medical head of the Accident and Emergency Department. Since 2002 he has been doing research mainly on the upper extremities, especially the scaphoid bone.

D. Ootes, M.S.

Daan Ootes is a medical student.

F.J.P. Beeres M.D., PhD.

Frank Beeres is a third year resident at the Medical Centre Haaglanden.

S.A.G. Meylaerts M.D., PhD.

Sven Meylaerts is a trauma surgeon at the Medical Centre Haaglanden and consultant for the Accident and Emergency Department.

I.B. Schipper M.D., PhD.

Prof. Dr. Inger Schipper is a trauma surgeon at the Leids University Medical Centre.

## Authors' contributions

SR: collected the data, put the conclusions together and drafted the manuscript.

DO: helped to find all the articles together, found the highlights, and drafted a part of the manuscript.

FB: particepated in the design of the study helped with the statistics.

SM: participated in the design of the study.

IS: conceived of the study and helped with the final manuscript.
